# Selenogenome and AMPK signal insight into the protective effect of dietary selenium on chronic heat stress-induced hepatic metabolic disorder in growing pigs

**DOI:** 10.1186/s40104-021-00590-2

**Published:** 2021-06-12

**Authors:** Yan Liu, Jiayong Tang, Ying He, Gang Jia, Guangmang Liu, Gang Tian, Xiaoling Chen, Jingyi Cai, Bo Kang, Hua Zhao

**Affiliations:** 1 Animal Nutrition Institute, Sichuan Agricultural University, Chengdu, 611130 Sichuan China; 2 Key Laboratory for Animal Disease-Resistance Nutrition, Ministry of Education, Huimin Road, Wenjiang District, Chengdu, 611130 Sichuan China; 3 College of Animal Science and Technology, Sichuan Agricultural University, Chengdu, 611130 Sichuan China

**Keywords:** Chronic heat stress, Hepatic metabolism, HMSeBA, Pigs, Selenoprotein

## Abstract

**Background:**

Chronic heat stress (CHS) disrupts hepatic metabolic homeostasis and jeopardizes product quality of pigs. Selenium (Se) may regulate the metabolic state through affect selenoprotein. Thus, we investigate the protective effect of dietary hydroxy-4-methylselenobutanoic acid (HMSeBA) on CHS induced hepatic metabolic disorder in growing pigs, and the corresponding response of selenoprotein.

**Methods:**

Forty crossbreed growing pigs were randomly assigned to five groups: control group raised in the thermoneutral environment (22 ± 2 °C) with basal diet; four CHS groups raised in hyperthermal condition (33 ± 2 °C) with basal diet and supplied with 0.0, 0.2, 0.4, and 0.6 mg Se/kg HMSeBA, respectively. The trial lasted 28 d. The serum biochemical, hepatic metabolism related enzyme, protein and gene expression and 25 selenoproteins in liver tissue were determined by real-time PCR, ELISA and western blot.

**Results:**

CHS significantly increased the rectal temperature, respiration rate, serum aspartate aminotransferase (AST) and low-density lipoprotein cholesterol (LDL-C) of pigs, up-regulated hepatic heat shock protein 70 (HSP70) and induced lower liver weight, glycogen content, hepatic glucokinase and glutathione peroxidase (GSH-Px). The CHS-induced liver metabolic disorder was associated with the aberrant expression of 6 metabolism-related gene and 11 selenoprotein encoding genes, and decreased the protein abundance of GCK, GPX4 and SELENOS. HMSeBA improved anti-oxidative capacity of liver. 0.4 or 0.6 mg Se/kg HMSeBA supplementation recovered the liver weight, glycogen content and rescue of mRNA abundance of genes related to metabolism and protein levels of GCK. HMSeBA supplementation changed expressions of 15 selenoprotein encoding genes, and enhanced protein expression of GPX1, GPX4 and SELENOS in the liver affected by CHS. CHS alone showed no impact while HMSeBA supplementation increased protein levels of p-AMPKα in the liver.

**Conclusions:**

In summary, HMSeBA supplementation beyond nutrient requirement mitigates CHS-induced hepatic metabolic disorder, recovered the liver glycogen content and the processes that are associated with the activation of AMPK signal and regulation of selenoproteins in the liver of growing pigs.

**Supplementary Information:**

The online version contains supplementary material available at 10.1186/s40104-021-00590-2.

## Background

As global warming intensifies, heat waves become more frequent and longer in duration [1]. Heat stress (HS) has been a common hazard affecting livestock production, which makes billions economic of losses in profit annually to livestock production [2]. The biological response to HS can be divided into acute and chronic phases, the acute phase lasting hours to a few days and the chronic phase lasting several days to weeks [3]. Chronic heat stress (CHS) lead to dysregulation of energy balance and metabolism [4], which caused the decreased quality of livestock products.

Pigs are particularly prone to HS due to thick layers of subcutaneous adipose tissue and lack of functional sweat glands [5]. Previous studies have shown that pigs reared in hyperthermal conditions typically had lower skeletal muscle weight, higher fat tissue mass and poorer meat quality [6, 7], and those responses are associate with the alternation of several metabolic parameters [8, 9]. Liver is a key metabolic organ that regulates the whole-body metabolism in animals and humans. Hepatic injury is a common clinical feature of HS, which leads to elevation of serum aspartate aminotransferase (AST), alanine transaminase (ALT) [10, 11]. It has been reported that CHS significantly reduced liver weight and altered proteomic-associated oxidative response, immune defense and metabolism [12].

Selenium (Se) is an essential micronutrient for humans and animals, and dietary Se supplementation relieves the HS-induced negative effects in intestinal and serum metabolism of pigs through the enhanced antioxidant capacity [13, 14]. Additionally, Se supplementation mitigates HS-induced injury in IPEC-J2 cells [15] and LPS-induced immunological stress in mice [16]. In addition to potentiate antioxidant defenses, Selenium is involved in the regulation of carbohydrate, protein and lipid metabolism [17, 18]. The biological function of Se is mainly mediated by selenoproteins [8]. Currently, 25 selenoprotein encoding genes have been identified in the porcine species [19], and several selenoproteins are involved in metabolic regulations [20]. Although the specific mechanisms are complex and remain unclear, the alternation of *GPX1*, *GPX4*, *SELENOH*, *SELENOP*, *SELENOS*, *DIO1* and *TXNRD1* are associated with gene expression related to glucose metabolism (*INSR*, *IRS1*, *AKT*, *PCK2* and *GCK*), lipogenesis (*FOXO1*, *FAS*, *ACC1* and *SREBP1*), and protein synthesis pathway (*mTOR*, *4E-BP1* and *RPS6*/*S6*) [17, 18, 21–25].

Taken together, studies have revealed that HS caused damage to the metabolic homeostasis [26] and Se supplementation alleviates various types of stress on animals [13]. However, the interaction of dietary Se supplementation and CHS on biomarkers related to liver metabolic function remains unclear and intriguing. The pig model offers unique advantages for the study of human nutrition and medicine due to their great similarities in digestive systems, nutrition metabolism, and physiological response to stress [27]. Hydroxy-4-methylselenobutanoic acid (HMSeBA) is a new organic Se with higher bioavailability [28]. Therefore, the pig CHS model was developed to investigate: 1) the protective effect of HMSeBA on alleviating the hepatic metabolic dysfunction induced by CHS, and 2) the possible mechanism that linked the alleviation of HMSeBA to the response of selenoproteins in the liver.

## Methods

### Animals, experiment design and management

Total of 40 crossbreed castrated boars (Landrace × Yorkshire) × Duroc, aged 14 weeks with average body weight of 49.64 ± 2.48 kg, were randomly divided into 5 treatments with 8 replicates and 1 pig per replicate (*n* = 8). The control group (CON) raised in a thermoneutral environment (22 °C) and fed on basal diet with no additional Se; the following four treatment groups were subjected to HS (33 ± 2 °C) with basal diet supplemented: 0.0 mg Se/kg (CHS), 0.2 mg Se/kg (CHS + 0.2HMSeBA), 0.4 mg Se/kg (CHS + 0.4HMSeBA), and 0.6 mg Se/kg (CHS + 0.6HMSeBA). The basal diet was formulated according to the National Research Council (2012) and meet the requirements of 50–75 kg class of pigs.

All pigs were housed in individual pen in the artificial climate chamber equipped with a climate control that allows the setting and control of the temperature and relative humidity. The environment temperature was gradually increased and kept at 27 °C on d 1, 28 °C on d 2. Thereafter, the temperature was kept at 33 ± 2 °C, the relative humidity (RH) was 77.05% ± 2.84% and the temperature-humidity index (THI) was 78.05 ± 4.37 in CHS groups while the temperature was maintained at 22 ± 2 °C, RH was 74.42% ± 2.32% and THI was 67.80 ± 0.88 in CON group, until the end of the trial. Pigs were free access to water and feed, and the trial lasted for 4 weeks. The rectal temperature (RT) and respiration rate (RR) of pigs were monitored at 09:00, 13:00 and 16:00 individually and weekly with a mercury thermometer and a mechanical counter as previous described [29].

### Blood and tissue collection

At d 28 after an overnight fast, the body weight of all pigs was recorded and six pigs with a body weight close to the average of each group were selected and the blood were collected in anticoagulant-free tubes from the jugular vein, and kept on the ice and centrifuged at 2,500×*g* for 10 min at 4 °C, then the serum was separated immediately and refrigerated at − 20 °C to analysis. Pigs were sedated by electrical stunning and slaughtered by manual exsanguination, and livers were separated and weighed and the liver index was calculated as the percentage of body weight. Liver samples were collected and rapidly freeze in liquid nitrogen and stored at − 80 °C for biochemical and molecular analyses.

### Selenium deposition and glycogen content in liver

The total Se concentration in liver was determined with a hydride generation flame atomic fluorescence spectrometer (AFS-3100, Hai Guang instrument, China) based on the national food safety standard of China (GB 5009.93–2010), and calculated according to protocol described in previous study [30]. The liver glycogen content was assessed with the commercial kits (Jiancheng Bioengineering, China) according to the manufacturer’s instructions.

### Serum biochemistry and hormone analyses

Serum alanine transaminase (ALT), aspartate aminotransferase (AST), total bile acid (TBA), total protein (TP), glucose (GLU), total triglycerides (TG), cholesterol (CHO), low-density lipoprotein cholesterol (LDL-C), high-density lipoprotein cholesterol (HDL-C) and non-esterified acid (NEFA) were measured using an automatic biochemistry analyzer (3100, HITACHI, Japan). Serum triiodothyronine (T3), tetraiodothyronine (T4) and fasting insulin (F-insulin) was analyzed with the commercially available radioimmunoassay kits (Beijing North Institute of Biological Technology, China). The procedures followed the manufacturers’ instructions. All measurements were conducted in duplicate.

### Antioxidant and metabolic enzyme analyses

Liver homogenates were prepared as previously described by our group [31]. The protein was quantified by the BCA protein assay kit (Jiancheng Bioengineering, China). Glutathione peroxidase (GSH-Px), total superoxide dismutase (T-SOD), total antioxidant capability (T-AOC) and malondialdehyde (MDA) were measured by colorimetric assay using commercial kits (Jiancheng Bioengineering, China). The activity of glucokinase (GCK), phosphoenolpyruvate carboxykinase (PEPCK) and fatty acid synthase (FAS) were measured using a commercial enzyme-linked immunosorbent assay (ELISA) kits (Meimian, China) according to the manufacturer’s instructions. For each measurement, the experiments were performed in triplicate at one occasion.

### Real-time PCR analyses

Total RNA of liver was isolated using the Trizol reagent (Invitrogen, USA) according to the manufacturer’s instructions and the reverse transcription was performed using the PrimeScript RT reagent kit (Takara, China). Primers (Supplementary Table 1) for 12 metabolism-related genes, 25 selenoprotein encoding genes and 2 reference genes (*β-ACTIN* and *GAPDH*) were designed with Primer Express 3.0 (Applied Biosystems, USA). Quantitative real-time PCR (Q-RT-PCR) was performed on QuantStudio 6 Flex system (Applied Biosystems, USA) using SYBR Premix Ex Taq™ II reagents (No. RR820A, Takara, China) as described previously [22, 31, 32].

### Western blot analyses

Liver total protein extracts were prepared using RIPA lysis buffer [50 mmol/L Tris-HCl (pH 7.4), 150 mmol/L NaCl, 0.25% sodium deoxycholate, 1% NP-40, 1 mmol/L EDTA, 10 μL/mL protease inhibitor, 10 μL/mL phosphatase inhibitor 3, 10 μL/mL 100 μmol/L Na_3_VO_4_, 10 μL/mL 10 mg/mL PMSF], and measured the protein concentration using a BCA kit (Jiancheng Bioengineering, China). Fixed protein amounts were electrophoresed using 12% SDS-PAGE gel and blotted onto PVDF membrane (Millipore, USA). The membranes were blocked and immunoblotted with primary antibodies against target protein HSP70 (1:5,000; ab5439; Abcam), AMPKα (1:1,000; #5831; Cell Signaling Technology), p-AMPKα (1:1,000; #2535; Cell Signaling Technology), GPX1 (1:1,000; 616,958; Zen BioScience), GPX4 (1:2,000; 513,309, Zen BioScience), SELENOS (1:1,000, 15,591–1-AP, Proteintech Group) and β-ACTIN (1:5,000; MAB1501; Millipore), respectively. Then incubated with corresponding secondary antibodies (horseradish peroxidase-linked goat anti-rabbit or mouse IgG). Autoradiography and chemiluminescence with an enhanced chemiluminescence system (Millipore, USA) was applied to detect and quantify the signal. Image Lab™ software system (Bio-Rad, USA) was used to analyze the densitometric of western blot bands. The ratio of target protein to β-actin protein represented the relative abundance of each target protein.

### Statistical analysis

The experiment was the complete random design (CRD) and applied the one-way structure treatment design. Analysis of rectal temperature and respiration rate during the experiment was performed using one-way analysis of variance (ANOVA) on d 0, 7, 14, 21, and 28 of the trial. The effect of different treatment was analysis using PROC MIXED of SAS 9.2 (SAS Institute, 2003). The Tukey test was used to adjust for multiple treatment comparisons using the LSMEAN statement of SAS 9.2 (SAS Institute, 2003) with letter groupings obtained using the SAS pdmix800 macro (Saxton, 1998) [33]. The normality and homogeneity of variances were evaluated by Shapiro-Wilk W test and Levene’s test using the UNIVARIATE and HOVTEST statement, respectively. For different statistical test, significance was declared at *P* ≤ 0.05 or highly significance at *P* ≤ 0.01, unless otherwise stated.

## Results

### Rectal temperature and respiration rate of growing pigs

**Fig. 1:**
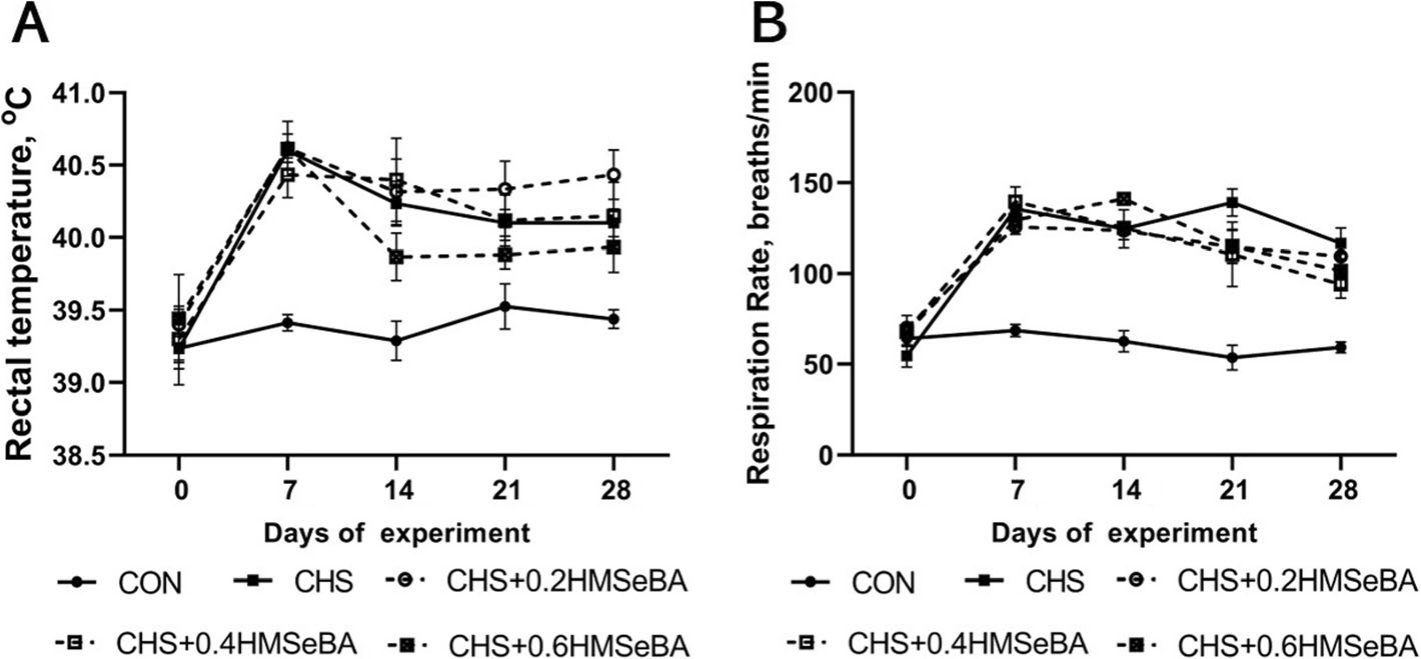
Effects of chronic heat stress and HMSeBA supplementation on rectal temperature (**a**) and respiration rate (**b**) of growing pigs. The *P*-value of ANOVA analysis on d 0, 7, 14, 21, and 28 of experiment were 0.927, < 0.01, < 0.01, < 0.01, and < 0.01 for rectal temperature and 0.269, < 0.01, < 0.01, < 0.01, and < 0.01 for respiration rate (*n* = 8)

To evaluate the effects of CHS on growing pigs, the rectal temperature and respiration rate were monitored every week. As expected, rectal temperature and respiration rate increased significantly in response to CHS at d 7, 14, 21 and 28 of the trial HMSeBA supplementation exhibited limited effect (*P* > 0.05) on the rectal temperature and respiration rate of pigs, however pigs received 0.6 mg Se/kg HMSeBA tended to have lower rectal temperature at d 14, 21 and 28, also pigs in three HMSeBA supplementation groups had a relative lower respiration rate at d 21 and 28 (Fig. 1a and b).

### Effects of CHS and HMSeBA supplementation on liver weight, index, glycogen content and se concentration

**Fig. 2:**
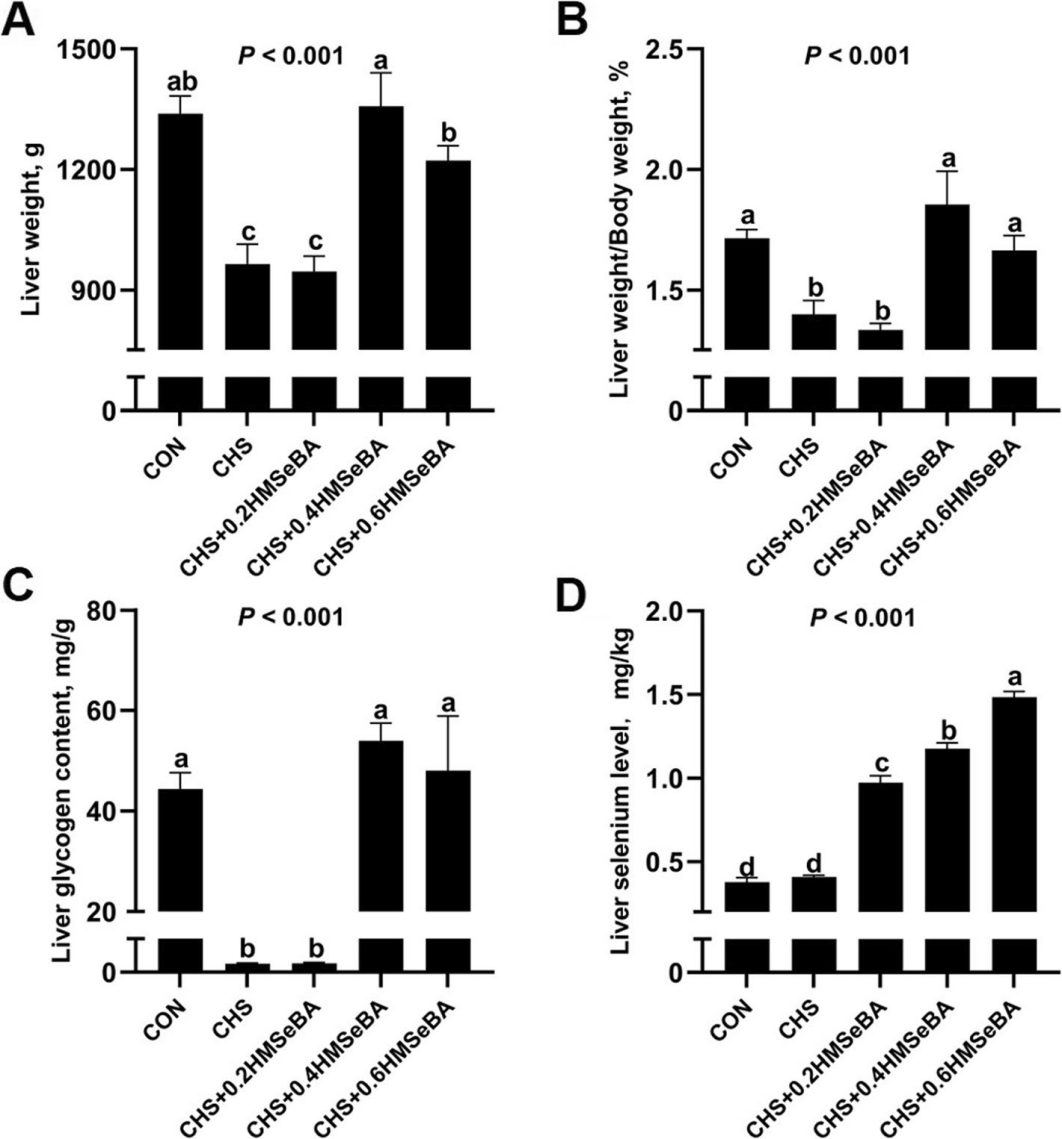
Effects of chronic heat stress and HMSeBA supplementation on liver weight (**a**), liver weight/body weight (**b**), liver glycogen content (**c**) and liver Se concentration (**d**) of growing pigs. The results were expressed as mean ± SEM (*n* = 6). Different letters denote significant differences (*P* < 0.05)

We investigated the effect of CHS on liver mass and Se deposition (Fig. 2). CHS decreased (*P* < 0.001) the absolute and relative liver weight and glycogen content. Dietary HMSeBA displayed a protective effect and 0.4 and 0.6 mg Se/kg HMSeBA effectively recovered the liver weight, liver weight index and glycogen content to normal level (*P* > 0.05) (Fig. 2a and b). CHS alone did not affect Se concentration, while dietary HMSeBA supplementation showed a dose-dependent increase in Se deposition in liver (Fig. 2c).

### Hepatic HSP70 abundance of growing pigs

**Fig. 3:**
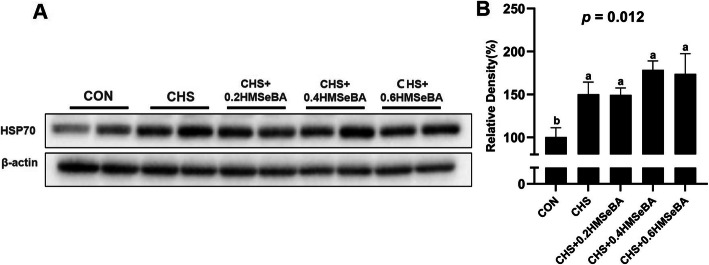
Effects of chronic heat stress and HMSeBA supplementation on the protein expression of HSP70 in liver. The results were expressed as mean ± SEM (*n* = 6). Different letters denote significant differences (*P* < 0.05)

The CHS affected the protein abundance of HSP70 in liver of pigs (Fig. 3). As expected, CHS significantly up-regulated the protein abundance of HSP70 in liver, which indicated pigs were suffered with heat stress. 0.4 and 0.6 mg Se/kg HMSeBA supplementation numerically increased HSP70 abundance compared with CHS groups.

### Effects of CHS and HMSeBA supplementation on hepatic antioxidant variables

**Fig. 4:**
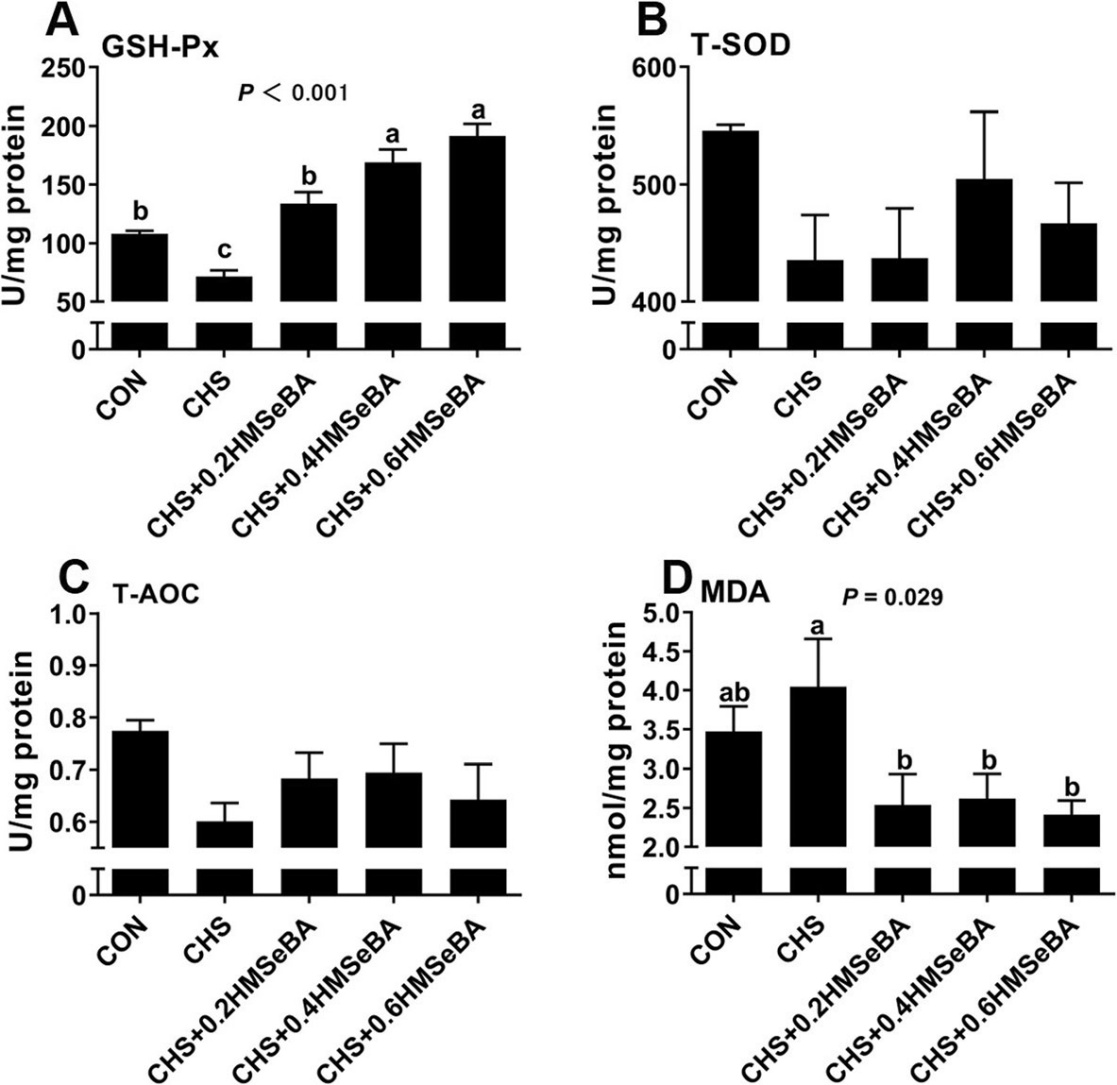
Effects of chronic heat stress and HMSeBA supplementation on GSH-Px (**a**), T-SOD (**b**), T-AOC (**c**) and MDA (**d**) concentration in liver. The results were expressed as mean ± SEM (*n* = 6). Different letters denote significant differences (*P* < 0.05)

We investigated the effect of HMSeBA supplementation on antioxidant measurements in liver of pigs under CHS (Fig. 4). CHS compromised the hepatic antioxidant by decreasing (*P* < 0.05) GSH-Px (Fig. 4a) and numerically increasing MDA levels (Fig. 4d). Although no statistical difference, CHS decreased (*P* > 0.05) T-SOD and T-AOC in liver. HMSeBA supplementation exhibited protective effect, which enhanced (*P* < 0.05) the GSH-Px activity in a dose dependence manner, and effectively decreased (*P* < 0.05) the MDA level in liver under CHS. Beyond this, HMSeBA supplementation elevated (*P* > 0.05) T-SOD and T-AOC in values in liver of pigs under CHS.

### Effects of CHS and HMSeBA supplementation on serum biochemical and hormone

We detected the effect of CHS on blood biochemical measures and endocrine (Table 1). Pigs subjected to CHS had higher (*P* < 0.05) serum TBA, LDL-C, AST and lower (*P* < 0.05) serum F-insulin and T3, and the serum T4 also tended to decrease (*P* < 0.1). HMSeBA supplementation moderately ameliorated (*P* < 0.05) the negative effect of CHS on serum AST and LDL-C. As shown in Table 1, 0.4 and 0.6 mg Se/kg HMSeBA returned (*P* < 0.05) serum AST activity to control level and 0.6 mg Se/kg HMSeBA reversed (*P* < 0.05) the serum LDL-C in pigs under CHS condition. Although CHS affected serum TBA, F-insulin, T3 and T4 concentration, dietary HMSeBA supplementation exhibited limited (*P* > 0.05) impact on those biochemical indicators. CHS or dietary HMSeBA showed no impact (*P* > 0.05) on serum ALT, GLU, TG, CHO, HDL-C and NEFA.

**Table 1:** Effects of chronic heat stress and HMSeBA supplementation on serum biochemical and endocrine parameters of growing pigs

Parameters	CON	CHS	CHS+ 0.2HMSeBA	CHS+ 0.4HMSeBA	CHS+ 0.6HMSeBA	ANOVA *P*-value
ALT, U/L	45.8 ± 3.5	48.0 ± 2.6	48.5 ± 4.7	47.2 ± 5.0	42.7 ± 1.6	0.807
AST, U/L	25.4 ± 2.0	38.5 ± 4.8	41.5 ± 2.7	35.0 ± 5.0	33.8 ± 3.4	0.101
TBA, μmol/L	25.7 ± 3.0^b^	56.1 ± 6.4^a^	48.9 ± 6.7^a^	56.0 ± 6.9^a^	55.6 ± 8.8^a^	0.017
F-insulin, μIU/mL	15.10 ± 2.63^a^	10.08 ± 0.98^b^	9.94 ± 0.70^b^	9.80 ± 0.76^b^	9.32 ± 0.69^b^	0.036
T3, ng/mL	0.65 ± 0.08^a^	0.45 ± 0.08^b^	0.42 ± 0.04^b^	0.34 ± 0.03^b^	0.39 ± 0.03^b^	0.014
T4, ng/mL	50.72 ± 2.31	37.08 ± 5.00	39.10 ± 3.25	34.89 ± 3.27	39.67 ± 1.02	0.055
GLU, mmol/L	5.30 ± 0.44	5.54 ± 0.15	5.06 ± 0.44	5.25 ± 0.42	5.67 ± 0.36	0.801
TG, mmol/L	0.52 ± 0.04	0.49 ± 0.03	0.53 ± 0.03	0.51 ± 0.03	0.54 ± 0.07	0.929
CHO, mmol/L	3.04 ± 0.11	3.28 ± 0.13	3.17 ± 0.16	3.14 ± 0.17	2.98 ± 0.13	0.597
LDL-C, mmol/L	1.04 ± 0.04^c^	1.36 ± 0.06^a^	1.27 ± 0.06^ab^	1.37 ± 0.08^a^	1.13 ± 0.05^bc^	0.002
HDL-C, mmol/L	0.80 ± 0.03	0.74 ± 0.04	0.74 ± 0.03	0.68 ± 0.05	0.70 ± 0.05	0.318
NEFA, mmol/L	0.20 ± 0.05	0.28 ± 0.19	0.27 ± 0.15	0.55 ± 0.25	0.36 ± 0.26	0.753

*ALT* alanine transaminase, *AST* aspartate aminotransferase, *TBA* total bile acid, *F-insulin* fasting insulin, *GLU* glucose, *TG* total triglycerides, *CHO* cholesterol, *LDL-C* low-density lipoprotein cholesterol, *HDL-C* high-density lipoprotein cholesterol, *NEFA* non-esterified acid, *T3* triiodothyronine, *T4* tetraiodothyronine. The results were expressed as mean ± SEM (*n* = 6). Values within a row with different superscripts differ (*P* < 0.05)

### Effects of CHS and HMSeBA supplementation on hepatic metabolic enzyme activity, metabolism related gene mRNA and protein expression

**Fig. 5:**
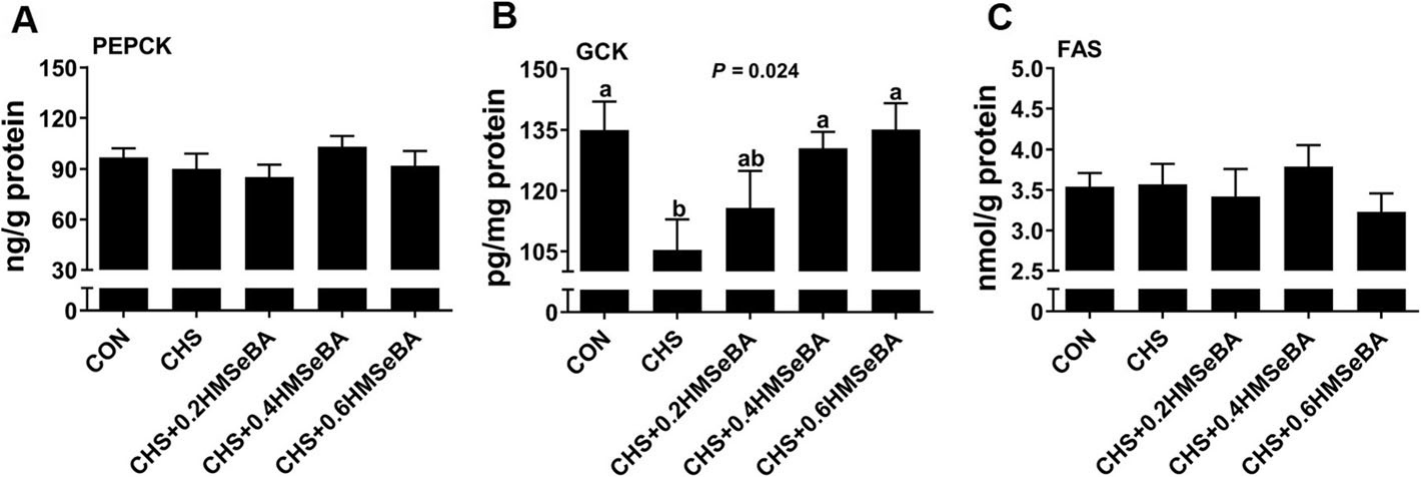
Effects of chronic heat stress and HMSeBA supplementation on PEPCK (**a**), GCK (**b**) and FAS (**c**) concentration in liver. The results were expressed as mean ± SEM (*n* = 6). Different letters denote significant differences (*P* < 0.05)

We assessed 3 hepatic enzymes related to liver metabolism (Fig. 5). CHS disturbed hepatic glucose metabolism by decreasing (*P* < 0.05) the GCK level while had limited impact (*P* > 0.05) on PEPCK and FAS. HMSeBA supplementation recovered (*P* < 0.05) the liver GCK levels in a dose dependence manner and 0.4 and 0.6 mg Se/kg HMSeBA recovered the liver GCK levels to normal levels. Dietary HMSeBA had no effect on hepatic PEPCK and FAS level (Fig. 5a and c).

**Fig. 6:**
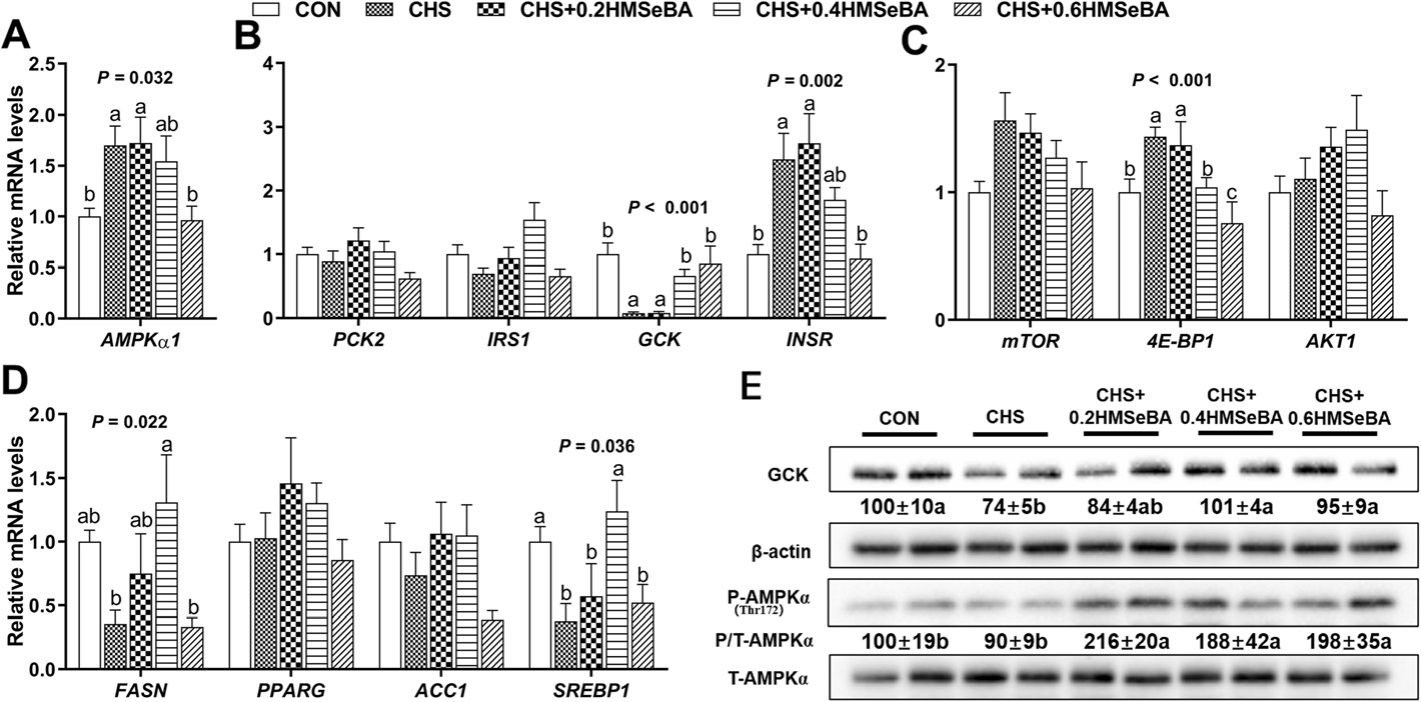
Effects of chronic heat stress and HMSeBA supplementation on expression of *AMPKα1* (**a**), genes related to glucose (**b**), protein (**c**) and lipid metabolism (**d**) and protein level of p-AMPKα1 and GCK (**e**) in liver. The results were expressed as mean ± SEM (*n* = 6). Different letters denote significant differences (*P* < 0.05)

We further investigated the response of mRNA levels of 12 metabolic related genes to HMSeBA in liver of pigs under CHS. The results showed that CHS up-regulated (*P* < 0.05) the mRNA levels of *AMPKα1*, *4E-BP1* and *INSR* (Fig. 6a, b and c), down-regulated (*P* < 0.05) mRNA levels of *GCK*, *FAS* and *SREBP1* (Fig. 6c and d) and exhibited no effect (*P* > 0.05) on expression of *mTOR*, *AKT1*, *PCK2*, *IRS1*, *PPARG* and *ACC1*. HMSeBA supplementation effectively prevented (*P* < 0.05) the up-regulation of *AMPKα1*, *4E-BP1* and *INSR* by CHS in a dose dependent manner. Meanwhile, HMSeBA supplementation reversed (*P* < 0.05) the down-regulation effect of CHS on *GCK*, *FAS* and *SREBP1*, among them*,* 0.4 and 0.6 mg Se/kg HMSeBA recovered the mRNA abundance of *GCK*, and 0.4 mg Se/kg HMSeBA recovered the mRNA abundance of *FAS* and *SREBP1* to normal levels. Other than that, dietary HMSeBA supplementation showed no impact on expressions of *mTOR*, *AKT1*, *PCK2*, *IRS1*, *PPARG* and *ACC1* in liver of pigs under CHS.

We investigated protein expression of GCK in liver, the results showed that CHS inhibited (*P* < 0.05) the protein expression of GCK, and the decrease of GCK protein level was reversed (*P* < 0.05) by dietary supplementation with 0.4 and 0.6 mg Se/kg HMSeBA (Fig. 6e). AMPKα is a key protein related to metabolic signal pathway. Although CHS exposure exhibited limited impact (*P* > 0.05) on the protein expression of p-AMPKα, three levels of dietary HMSeBA supplementation increased (*P* < 0.05) its proteins abundance (Fig. 6e).

### Effects of CHS and HMSeBA supplementation on mRNA and protein expression of selenoproteins

**Fig. 7:**
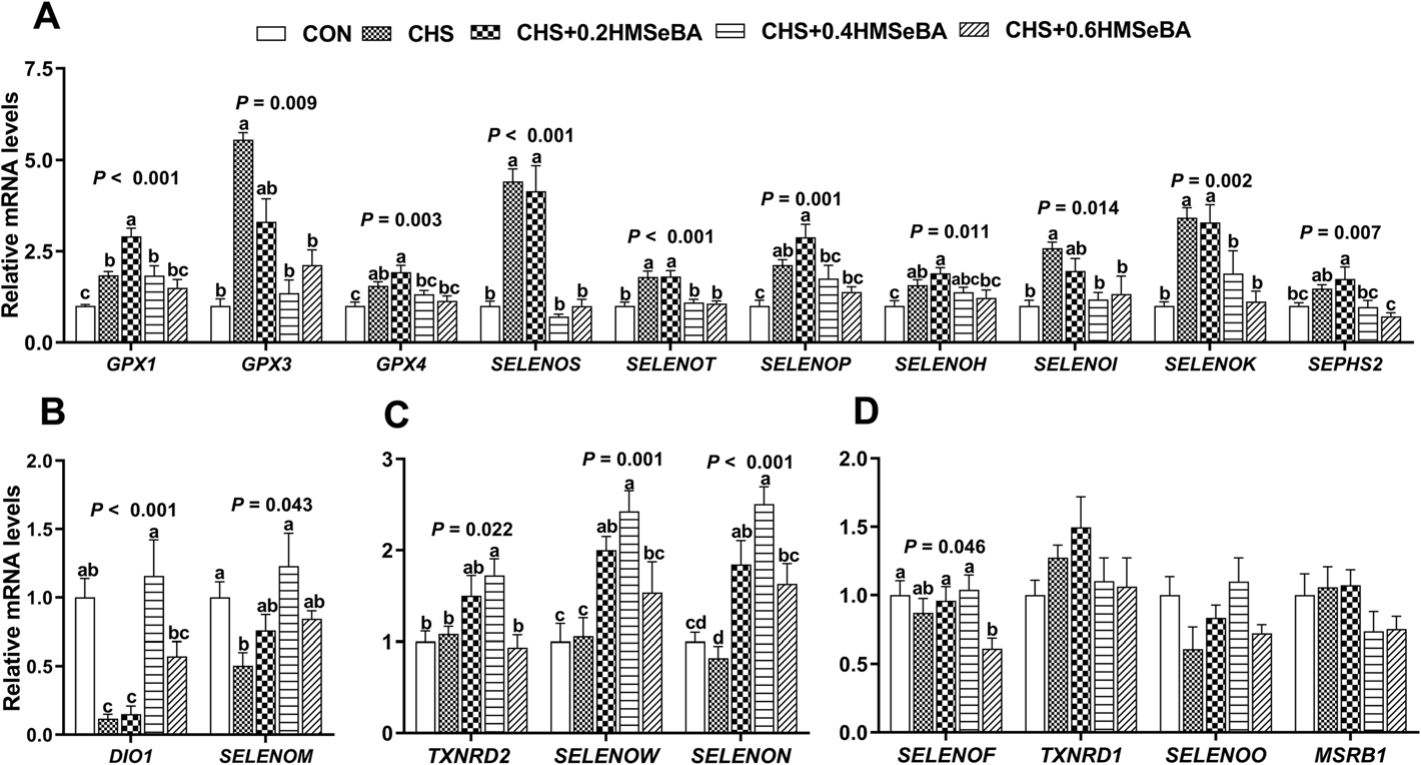
Effects of chronic heat stress and HMSeBA supplementation on expression of selenoprotein encoding genes in liver. The results were expressed as mean ± SEM (*n* = 6). Different letters denote significant differences (*P* < 0.05)

mRNA abundance of 25 selenoprotein encoding genes in liver of pigs were explored (Fig. 7). CHS increased (*P* < 0.05) mRNA expression of 10 selenoprotein genes (*GPX1*, *GPX3*, *GPX4*, *SELENOS*, *SELENOT*, *SELENOP*, *SELENOH*, *SELENOI*, *SELENOK,* and *SEPHS2*) (Fig. 7a), decreased (*P* < 0.05) mRNA expression of *DIO1* and *SELENOM* (Fig. 7b), and exhibited no effect on expression of *TXNRD2*, *SELENOW* and *SELENON* (Fig. 7c). Dietary HMSeBA supplementation exhibited impact on expression of selenoprotein encoding genes in liver of pigs under CHS, which decreased (*P* < 0.05) expression of *GPX3*, *GPX4*, *SELENOS*, *SELENOT*, *SELENOP*, *SELENOH*, *SELENOI*, *SELENOK*, *SEPHS2*, *DIO1*, and *SELENOM*)(Fig. 7a) and increased (*P* < 0.05) the mRNA abundance of *DIO1* and *SELENOM* (Fig. 7b). CHS did not affect the expression of *TXNRD2*, *SELENOW* and *SELENON*, while dietary 0.2 or 0.4 mg Se/kg HMSeBA supplementation increased (*P* < 0.05) their expression in liver of pigs under CHS (Fig. 7c). Additionally, CHS or dietary HMSeBA did not affected expression of *SELENOF*, *TXNRD1*, *SELENO* and *MSRB1.* Taken together, HMSeBA supplementation alleviated the impact of CHS on expression of selenoprotein encoding genes in liver of pigs, 0.4 or 0.6 mg Se/kg dietary HMSeBA supplementation exhibited better recovery effect based on the expression of these selenoprotein encoding genes, which shared similar mRNA profiles compared with that of the control.

**Fig. 8:**
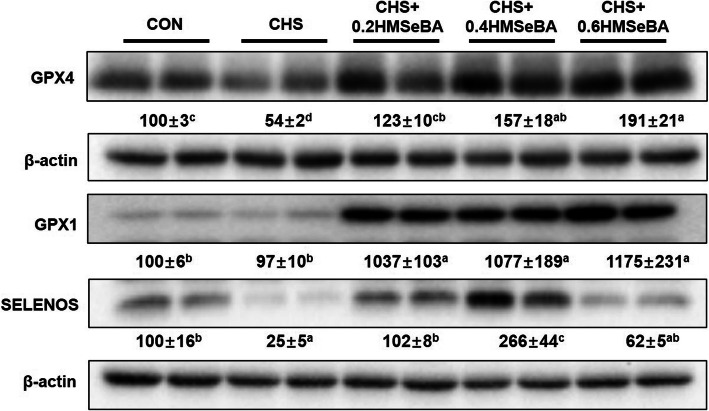
Effects of chronic heat stress and HMSeBA supplementation on the protein expression of GPX1, GPX4 and SELENOS in liver. The results were expressed as mean ± SEM (*n* = 4 or 6). Different letters denote significant differences (*P* < 0.05)

We also investigated protein expression of 3 selenoproteins (Fig. 8). CHS affected expression of selenoproteins, which inhibited (*P* < 0.05) the protein expression of GPX4 and SELENOS. HMSeBA supplementation inhibited (*P* < 0.05) this CHS induced reduction and the protein expression of GPX4 was enhanced (*P* < 0.05) with the increased of HMSeBA supplementation. Also, the decreased SELENOS was reversed (*P* < 0.05) in CHS + 0.2HMSeBA group and enhanced (*P* < 0.05) expression in CHS + 0.4HMSeBA group. CHS did not affected the expression of GPX1, while three levels of dietary HMSeBA supplementation greatly increased (*P* < 0.05) its protein expression in liver of pigs under CHS.

## Discussion

CHS negatively affects animals in various aspects including physiology, oxidative and metabolism balance. In this study, it was hypothesized that HMSeBA supplementation may improve liver antioxidant capacity and mitigate liver metabolic dysfunction induced by CHS. As expected, all pigs housed in the hyperthermal environment showed a significantly higher RT and RR which are typical symptoms of CHS and have been recorded in several studies about the HS animal model [14, 34]. Similar to the results reported previously [14], compared with the CHS group, high dietary HMSeBA supplementation (0.6 mg Se/kg) numerically decreased the RT of pigs during the middle and late period of the trial, which may be related to the reduced total heat production in high Se supplementation group.

Heat shock proteins (HSPs) are ubiquitous and strongly induced by heat shock which is usually categorized according to their molecular weight [35]. Among the HSPs, HSP70 is frequently used to evaluate HS response, which is considered as a cellular thermometer [36]. As expected, in this study the hepatic HSP70 protein abundance was increased in 4 CHS exposure groups, which is consistent with our previous study [15, 16]. HSP70 performs multiple roles which are important for maintaining cell survival during hyperthermia [35]. The up-regulation expression of the HSP70 helps restore unfolded or misfolded proteins to native conformation under HS condition [37]. Se supplementation effectively alleviates the decreased cell viability by HS and increases the protein abundance of HSP70 under HS in IPEC-J2 cells [15]. Similar to the previous study, 0.4 and 0.6 mg Se/kg HMSeBA supplementation numerically elevated the HSP70 protein level in the liver (Fig. 3), and this response may have potential beneficial effect for hepatic cells under CHS.

It is reported that CHS led to hepatic injury accompany the change in the expression of proteins that are mainly involved in oxidative stress response [12]. Consistently, CHS decreased hepatic GSH-Px activity and increased hepatic MDA level (Fig. 4). GSH-Px, T-SOD and T-AOC are important enzymes involved in the cell antioxidant system [16] and MDA is usually used as a biomarker to assess oxidative stress in an organism [38]. These results indicate that CHS weakened the antioxidant systems and led to oxidative stress response in the liver. The hepatic Se deposition and GSH-Px activity was elevated with the increased HMSeBA supplementation, and the MDA content was also decreased under CHS (Figs. 2 and 4). As we know, tissue GPX-Px is always used as an indicator of body selenium status. Therefore, it is not difficult to understand the relationship between the live Se concentration and hepatic GPX-Px activity in current study. In summary, HMSeBA supplementation beyond requirement relieves the hepatic antioxidant damage of pigs suffered from CHS.

In the current study, CHS led to significant decrease in liver weight and index of growing pigs (Fig. 2a and b), which is consistently to the previous report in growing pigs [12]. Meanwhile, the hepatic glycogen was depleted in CHS pigs which implied morbid liver function. It has been reported that CHS induced liver injury occurs in parallel with serum biochemical abnormalities and metabolic dysfunction [4, 34]. It is unexpected that 0.4 and 0.6 mg Se/kg HMSeBA supplementation significantly increased the live glycogen content under CHS. Se ameliorates the chronic liver injury by altering the serum biochemical indices [39]. Although the information about the effects of Se on liver metabolic functions of pigs under CHS is limited, it has been credited that Se has insulin-mimetic and anti-diabetic properties [17], and selenite affects the expression of liver glucose metabolism enzymes in a diabetic rat model [40]. HS affects metabolic function, which decreases serum F-insulin, T3 and T4 concentration and elevates the insulin sensitive [6, 9]. In this study, CHS led to an increase of serum AST, TBA, LDL-C, concentration and a decrease of serum F-insulin, T3, T4 and hepatic GCK level (Table 1; Fig. 5b). Serum ASL is an indicator of liver injury and the increase of serum ASL reflects the magnitude of liver damage [41]. Serum TBA is a highly sensitive marker for liver injury and dysfunction due to minor liver damage cause an increase of serum TBA [42]. LDL-C is the main resource of cholesterol from blood to the liver and liver is the essential organ in cholesterol synthesis and metabolism [43]. GCK is the first and rate-limiting step of glycolysis which catalyzes the conversion of glucose to glucose 6-phosphate in hepatocytes, more importantly, GCK participates in glycogen synthesis [44]. Our results suggest that severe liver injury and functional disruptions of substrates metabolism occurred in the liver. The liver metabolism function under CHS was partially normalized by Se. HMSeBA supplementation partially assuaged these negative reactions of CHS and mostly represented in the decrease of serum AST and LDL-C (Table 1) and an increase of hepatic GCK level (Fig. 5b). Also, the protective effects could be confirmed by the recovery of liver weight, liver index and glycogen content by 0.4 and 0.6 mg Se/kg HMSeBA supplementation (Fig. 2). Therefore, HMSeBA supplementation enhanced the glycogen synthesis in the liver of growing pigs under CHS.

We examined the expression profile of the genes related to hepatic energy homeostasis, protein synthesis, glucose and lipid metabolism. CHS up-regulated the mRNA expression of *AMPKα1*, *4E-BP1*, *INSR* and down-regulated mRNA expression of *FASN*, *SREBP1* and especially the *GCK* (Fig. 6). Under energy imbalance or stress, AMPK is activated by phosphorylation to cope with the adverse environments and switch on the catabolic pathway and inhibit anabolic [45]. Dephosphorylated 4E-BP1 binds to elF4E and inhibits protein translation function [46]. FASN and SREBP1 carry an important role in liver lipogenesis [18]. It is not difficult to understand the decreased mRNA expression of *FASN* and *SREBP1* under a negative energy balance in CHS, and appropriative HMSeBA supplementation may affect these two genes through the optimization hepatic energy metabolism. The increased mRNA expression of INSR may interpret the increased insulin sensitivity under HS [9]. Overall, the aberrant responses of these genes in the liver reflect the abnormal metabolic function of pigs under CHS. HMSeBA supplementation moderately recovered the expression of these affected genes, indicating a protective effect.

Se exerts important roles in energy metabolism [7, 18, 21], and the biological functions of Se are mainly mediated by selenoproteins. Expressions of nearly all porcine selenoprotein genes are responsive to dietary Se intakes of pigs in different tissues, and 9 hepatic selenoprotein encoding genes (*GPX1*, *GPX3*, *GPX4*, *TXNRD1*, *SELENOS*, *SELENOP, SELENOT, SELENOW* and *SELENOH*) of pigs are altered by Se deficiency or excess [18, 22, 23, 47–51]. In this study, selenoprotein encoding genes performed 4 patterns in the liver in response to CHS and Se supplementation.

Firstly, 10 selenoprotein encoding genes were up-regulated by CHS, while all of them were down-regulated by supplementation with 0.6 mg Se/kg HMSeBA, and 8 and 2 of them were down-regulated by 0.4 or 0.2 mg Se/kg HMSeBA (Fig. 7a). Among these affected genes, *GPX1*, *GPX3*, *GPX4*, *SELENOP* and *SELENOH* have been shown to be related to insulin signaling, glycolysis, gluconeogenesis, lipogenesis and protein synthesis pathway in pig tissues [18, 22, 23, 52]. The up-regulation of these genes under CHS is consistent with the previous study [53], and is associated with the decreased gene expression of *GCK*, *FASN* and SREBP1 in rat liver [19, 20, 54]. The increased *SELENOS* mRNA level is along with the up-regulation of *INSR* in rat liver [24]. The function of SELENOT, SELENOI and SELENOK in porcine remains unclear. Currently, SELENOI may be connected to the synthesis of steroid hormones and proteins for the plasma membrane of humans [55]. SELENOK and SELENOT are involved in redox metabolism regulation [56, 57]. Therefore, the abnormal up-regulation of these selenoproteins might be related to the CHS induced hepatic metabolism disorder. Among the above mentioned selenoprotein encoding genes affected by CHS, *GPX1*, *GPX3*, *GPX4*, *SELENOS*, *SELENOP* and *SELENOH* are up-regulated with the increased Se supplementation in liver [18, 19, 22, 47, 50]. The mRNA levels of *SELENOK*, *SELENOT*, and *SEPHS2* in liver or other organs of pigs or rodents are somewhat resistant to dietary Se deficiency or excess [19, 22, 49]. Additionally, in multiple porcine tissues (pituitary, spleen, and thyroid) both dietary Se deficiency and excess decrease the expression of *SELENOI* [22]. However, in current study, the expression of these hepatic selenoproteins decreased with the increase of dietary Se content under CHS. Our previous studies *in vitro* also found the similar phenomenon, HS up-regulates most selenoprotein encoding genes in IPEC-J2 cells [15] and differentiated C2C12 cells [53, 58]. The down-regulation of these genes may be a special hierarchical response of Se to alleviate the CHS induced hepatic damage and promoted normalization of metabolism function.

The second pattern was that CHS down-regulated the mRNA expression of *DIO1* and *SELENOM* in the liver, and HMSeBA supplementation partially increased the mRNA expression of these two genes (Fig. 7b). DIO1 is an oxidoreductase with SeCys residue in the active site and participate in thyroid hormone metabolism [59] and the decrease of mRNA abundance of *DIO1* is associated with insulin resistance, lipogenesis and protein synthesis in skeletal muscle or liver of pigs [18]. Se counter thyrotoxicity of Di-(2-ethylhexyl) phthalate (DEHP) via elevating DIO1, and elevates plasma T3 and T4 that was decreased by DEHP in rats [60]. Our results showed that CHS significantly decreased the serum T3 and T4, however, HMSeBA supplementation does not show significant effect under CHS, and this maybe an adaptive response to CHS. The hepatic *DIO1* mRNA expression response to CHS and HMSeBA supplementation may reflect a local organ reaction. Additionally, porcine *DIO1* transcripts is unaltered by dietary Se deficiency in the liver [22], and *DIO1* mRNAs levels decrease less than that of *GPX1* mRNA in a Se deficiency rodents, because *DIO1* has a relatively high hierarchy of Se status [61]. Therefore, the decreased *DIO1* expression in current study is mainly caused by CHS, and the restoration of endocrine and gene expression may be related to the systemic remission of CHS.SELENOM is a thiol-disulfide oxidoreductase and participates in the protection against superoxide and regulation of apoptosis [62]. The expression of porcine splenic *SELENOM *is up-regulated by a moderately high intake of dietary Se [49]. The down-regulation of this gene may reflect hepatic oxidative injury and metabolism dysfunction induced by CHS. 

The third pattern was that CHS had no effects on mRNA expression of *TXNRD2*, *SELENOW* and *SELENON*, while HMSeBA supplementation moderately enhances their mRNA expression and CHS + 0.4 HMSeBA group exhibited a higher profile (Fig. 7c). *TXNRD2* has been reported to protect cells from oxidant stress during embryogenesis [63]. *SELENOW* also has an antioxidant function and overexpression of SELENOW enhance the resistance of hamster ovary and lung cancer cells on H_2_O_2_ cytotoxicity [64] *SELENON* is ubiquitously expressed in muscle, brain, lung and fetal tissue. Although its specific biological function remains unclear, this selenoprotein plays a key role in the proliferation of fibroblast [65]. The expression of porcine splenic *SELENON* is up-regulated by a high dietary Se supplementation [49], and dietary Se deficiency decreases expression of hepatic *SELENOW* of pigs [22]. However, mRNA levels of *TXNRD2* in tissues or cells of pigs are not sensitive to dietary Se deficiency or excess [22, 49]. Even though the response of these three selenoproteins encoding genes in this study is a litter different from the results of previous studies on pigs and rats, which may reflect that the changed in the synthesis hierarchy of the selenoproteins in HS pig liver, and increased mRNA expression of these genes may contribute to the enhanced antioxidant capacity in the liver. Additionally, the response of *SELENOF*, *TXNRD1*, *SELENOO* and *MSRB1* were insensitive to CHS and HMSeBA supplementation (Fig. 7d).

Taken together, the alternation of the selenogenome and associated metabolism genes expression may be a symptom of lack of metabolic homeostasis under CHS. Dietary HMSeBA supplementation (0.4 or 0.6 mg Se/kg) recovered the most profiles of mRNA of selenoproteins in the liver of hyperthermia stressed pigs, which profiles were much similar to that of control pigs. These results suggested that Se alleviates the CHS induced metabolic disorder in the liver of growing pigs mainly by regulating the expression of selenoproteins.

For further determine the relationship between key selenoproteins and metabolism functions, the protein abundance of GPX1, GPX4 and SELENOS were detected. As shown in Fig. 8, CHS decreased the protein expression of GPX4 and SELENOS, and HMSeBA supplementation recovered or further increased the protein abundances of GPX4, SELENOS and GPX1. The selenoprotein response to CHS and dietary Se were inconsistent to the mRNA expression, which may result from the complicated regulation in the transcription, mRNA decay, translation, amino acid properties, and protein degradation [66, 67], and possibly other processes. HS is usually accompanied by severe oxidative stress [13]. GPX1 and GPX4 belong to antioxidant enzyme, the increase of their protein abundance suggests enhancement of hepatic antioxidant capacity in the stressed pigs. SELENOS is an important endoplasmic reticulum transmembrane protein, and overexpression of SELENOS enhances cells’ resistance to oxidative stress [68]. CHS decreased mRNA level, protein abundance (Fig. 6) and activity of hepatic GCK (Fig. 5). GCK catalyzes the conversion of glucose to G-6-P, which modulates glycogen synthesis in liver [44]. The rescued GCK in the liver suggest the mitigation of hepatic metabolic disorder. The protective effect of HMSeBA is mainly through regulation of selenoprotein. In current study, dietary HMSeBA enhanced the protein abundance of GPX1, which is positive correlation with the activity of GCK [20]. In addition, HMSeBA supplementation up-regulated p-AMPKα (Fig. 6e), and the elevation of GPX1 activity increases the hepatic p-AMPKα [18]. AMPK is activated by phosphorylation of the catalytic subunit then regulate the metabolic processes [69]. Therefore, dietary HMSeBA supplementation optimized the hepatic metabolic state under CHS through regulation of selenoproteins and the process is related to the activation of the AMPK signal.

## Conclusions

In summary, in present study, CHS causes metabolic disorders in the liver of growing pigs, which is accompanied by alteration of physiological parameters, depleted hepatic glycogen and aberrant expression of selenoprotein encoding genes and selenoprotein. HMSeBA supplementation alleviates the CHS-induced negative effects to the liver with the enhancement of antioxidant capability. More importantly, supplementation with dietary HMSeBA beyond nutrient requirements (0.4 and 0.6 mg/kg) effectively recovers the most profiles of mRNA and proteins of selenoproteins in the liver of pigs suffered from CHS, which profiles are much similar to that of control pigs accompanied by the hepatic glycogen content significantly recovered, thus corresponding hepatic metabolic disorder are alleviated. Especially, the protective effects of HMSeBA are associated with the activation of AMPK signal.

## Supplementary Information

**Additional file 1: Supplemental Table 1.** Primers used for the q-PCR of the target and reference genes.

## Data Availability

The datasets produced and/or analyzed during the current study are available from the corresponding author on reasonable request.

## Abbreviations

CHS: Chronic heat stress
Se: selenium
HMSeBA: Hydroxy-4-methylselenobutanoic acid
AST: Aspartate aminotransferase
LDL-C: Low-density lipoprotein cholesterol
ALT: Alanine transaminase
TBA: Total bile acid
TP: Total protein
GLU: Glucose
TG: Total triglycerides
CHO: Cholesterol
NEFA: Non-esterified acid
F-insulin: Fasting insulin
HSP70: Heat shock protein 70
T-SOD: Total superoxide dismutase
MDA: Malondialdehyde
GSH-Px: Glutathione peroxidase
T-AOC: Total antioxidant capability
GCK: Glucokinase
PEPCK: Phosphoenolpyruvate carboxykinase
FAS: Fatty acid synthase
AMPKα: AMP-activated protein kinase alpha
p-AMPKα: Phospho- AMP-activated protein kinase alpha
PCK2: Phosphoenolpyruvate carboxykinase 2
IRS1: Insulin receptor substrate 1
INSR: Insulin receptor
mTOR: Mammalian target of rapamycin
4E-BP1: 4 E-binding protein
AKT1: Serine/threonine-protein kinase
PPARG: Peroxisome proliferative activated receptor, gamma
ACC1: Acetyl coenzyme a carboxylase 1
SREBP1: Sterol-regulatory element binding protein 1
