# Supplementary material for: Selenogenome and AMPK signal insight into the protective effect of dietary selenium on chronic heat stress-induced hepatic metabolic disorder in growing pigs
Source: J Anim Sci Biotechnol. 2021 Jun 12;12:68. doi: 10.1186/s40104-021-00590-2 (PMC8196429; doi:10.1186/s40104-021-00590-2)
Supplement: Supplementary file 1 — Additional file 1: Supplemental Table 1. Primers used for the q-PCR of the target and reference genes. [file 40104_2021_590_MOESM1_ESM.docx]

**Supporting table 1.** Primers used for the q-PCR of the target and reference genes

| **Protein** | **Gene** | **Accession number** | **Primer sequence (from 5’ to 3’)** |
| --- | --- | --- | --- |
| **Selenoprotein encoding genes** | | | |
| DIO1 | *DIO1* | AY533206 | F: CATGGCCAAGAACCCTCACT  R: CCAGAAATACTGGGCACTGAAGA |
| DIO2 | *DIO2* | AY533207 | F: CGCTGCATCTGGAAGAGCTT  R: TGGAATTGGGTGCATCTTCA |
| DIO3 | *DIO3* | AY533208 | F: TGAAGTGGAGCTCAACAGTGATG  R: TGTCGTCAGACACGCAGATAGG |
| GPX1 | *GPX1* | AF532927 | F: GATGCCACTGCCCTCATGA  R: TCGAAGTTCCATGCGATGTC |
| GPX2 | *GPX2* | DQ898282 | F: AGAATGTGGCCTCGCTCTGA  R: GGCATTGCAGCTCGTTGAG |
| GPX3 | *GPX3* | AY368622 | F: CCGGTTCCTGTTTTCCAAATT  R: TGCACTGCAGGAAGAGTTTGAA |
| GPX4 | *GPX4* | NM_214407 | F: TGAGGCAAGACGGAGGTAAACT  R: TCCGTAAACCACACTCAGCATATC |
| GPX6 | *GPX6* | NM_001137607 | F: GAGCTGAAGCCTTTTGGTGTAGTT R: CTTTGCTGGTTCTTGTTTTCCA |
| MSRB1 | *MSRB1* | EF113597 | F: ATCCCTAAAGGCCAAGAATCATC  R: GGCCACCAAGCAGTGTTCA |
| SELENOF | *SELENOF* | EF178474 | F: ACAGCCCTGCCAAGCAGAT  R: AACAGGGAGGCTGGGTAACAC |
| SELENOH | *SELENOH* | HM018602 | F: TGGTGGAGGAGCTGAAGAAGTAC  R: CGTCATAAATGCTCCAACATCAC |
| SELENOI | *SELENOI* | NM_001244662.1 | F: GATGGTGTGGATGGAAAGCAA  R: GCCATGGTCAAAGAGTTCTCCTA |
| SELENOK | *SELENOK* | DQ372075 | F: CAGGAAACCCCCCTAGAAGAA  R: CTCATCCACCGGCCATTG |
| SELENOM | *SELENOM* | FJ968780 | F: CAGCTGAATCGCCTCAAAGAG  R: GAGATGTTTCATGACCAGGTTGTG |
| SELENON | *SELENON* | EF113595 | F: ACCTGGTCCCTGGTGAAAGAG  R: AGGCCAGCCAGCTTCTTGT |
| SELENOO | *SELENOO* | AK236851 | F: CTTCCGACCCCAGATGGAT  R: GGTTCGACTGTGCCAGCAT |
| SELENOP | *SELENOP* | EF113596 | F: AACCAGAAGCGCCAGACACT  R: TGCTGGCATATCTCAGTTCTCAGA |
| SELENOS | *SELENOS* | AY609646 | F: GAGGCAGAGGCACCTGGAT  R: CTGCTAAAGCCTCCTGTCGTTT |
| SELENOT | *SELENOT* | AY609428 | F: GGCTTAATAATCGTTGGCAAAGA  R: TGGCCCCATTGCCAGATA |
| SELENOV | *SELENOV* | GQ478346 | F: CACTGGTCGCCAATGGATTC  R: AGTGGCCAACGGAGAAAGC |
| SELENOW | *SELENOW* | NM_213977 | F: CACCCCTGTCTCCCTGCAT  R: GAGCAGGATCACCCCAAACA |
| SEPHS2 | *SEPHS2* | EF033624 | F: TGGCTTGATGCACACGTTTAA  R: TGCGAGTGTCCCAGAATGC |
| TXNRD1 | *TXNRD1* | AF537300 | F: GATTTAACAAGCGGGTCATGGT  R: CAACCTACATTCACACACGTTCCT |
| TXNRD2 | *TXNRD2* | GU181287 | F: TCTTGAAAGGCGGAAAAGAGAT  R: TCGGTCGCCCTCCAGTAG |
| TXNRD3 | *TXNRD3* | BX918808 | F: GTGCCCTACGTTTATGCTGTTG  R: TCCGAGCCACCAGCTTTG |
| **Metabolism-related genes** | | | |
| AMPKα1 | *AMPKα1* | NM_001167633.1 | F: TTGACTCGGCCCCATCCT  R: GTATGGCGTGCCCTTGGA |
| GCK | *GCK* | XM_013985832.2 | F: GTGGTGGCAATGGTGAATGAC  R: TCGGCGGTCTTCATAGTAGCA |
| PCK2 | *PCK2* | NM_001161753.1 | F: GCCCTTCTTCGGCTACAACTTT  R: CCTTGCGCCCCTCCAT |
| INSR | *INSR* | AF102858.1 | F: CCAAAGGCCAGCCAACACT  R: GGGAACGCAGGTAACTCTTTAAGTC |
| AKT1 | *AKT1* | NM_001159776.1 | F: AGAACCGCGTCCTCCAGAA  R: CGTGGGTCTGGAAGGAGTACTTC |
| SREBF1 | *SREBF1* | NM_214157.1 | F: GCTGAATAAATCCGCCGTCTT  R: CTGGTTGCTCTGCTGAAGGAA |
| ACC1 | *ACC1* | XM_021066238.1 | F: CAAGACCACCAACGCGAAA  R: GGCAAATGGGAGGCAATAAGA |
| PPARG | *PPARG* | NM_214379.1 | F: TGCCACAGGCTGAGAAGGA  R: GGGTTCAGCTGGTCGATATCAC |
| FASN | *FASN* | NM_001099930.1 | F: GTGGGTGTGAGCAGTTCTGATG  R: GCCCCTTGAAGTCAAAGAAGAAG |
| mTOR | *mTOR* | XM_003127584.6 | F: GGACACAAACACCAAAGGTAACAAG  R: GTGGTCCCCGTTTTCTTATGG |
| 4E-BP1 | *4E-BP1* | NM_001244225.1 | F: CCCCCTGCTTCCTCACTCA  R: TGGAGGTATCTGCTGGTGTTCA |
| **Housekeeping control genes** | | | |
| β-ACTIN | *β-ACTIN* | NM_007393.5 | F: ACCAGTTCGCCATGGATGAC  R: TGCCGGAGCCGTTGTC |
| GAPDH | *GAPDH* | GU214026.1 | F: GGGAAGCCCATCACCATCT  R: CGGCCTCACCCCATTTG |
